# Reasons given by general practitioners for non-treatment decisions in younger and older patients with newly diagnosed type 2 diabetes mellitus in the United Kingdom: a survey study

**DOI:** 10.1186/1472-6823-11-17

**Published:** 2011-10-28

**Authors:** Qiaoyi Zhang, Elizabeth Marrett, Kevin Jameson, Susanne Meiler, Michael J Davies, Larry Radican, Alan J Sinclair

**Affiliations:** 1 Merck Sharp & Dohme Corp., Whitehouse Station, NJ USA; 2 MSD UK Ltd., Hoddesdon, UK; 3 Kantar Health GmbH, München, Germany; 4 Beds & Herts Postgraduate Medical School, Luton, UK

## Abstract

**Background:**

Older patients with newly diagnosed type 2 diabetes mellitus are less likely to receive antihyperglycaemic therapy compared to their younger counterparts. The purpose of this study was to assess the reasons of general practitioners (GPs) for not treating younger and older patients with newly diagnosed type 2 diabetes mellitus with antihyperglycaemic agents.

**Methods:**

In a survey conducted between November 2009 and January 2010, 358 GPs from the United Kingdom selected reasons for not initiating antihyperglycaemic therapy in younger (< 65 years) and older (≥65 years) patients with newly diagnosed type 2 diabetes mellitus and untreated with any antihyperglycaemic agent for at least six months following diagnosis. Thirty-six potential reasons were classified into four major categories: *Mild hyperglycaemia*, *Factors related to antihyperglycaemic agents*, *Comorbidities and polypharmacy*, and *Patient-related reasons*. Reasons for non-treatment were compared between younger (n = 1, 023) and older (n = 1, 005) patients.

**Results:**

Non-treatment reasons related to *Mild hyperglycaemia *were selected more often by GPs for both younger (88%) and older (86%) patients than those in other categories. For older patients, *Factors related to antihyperglycaemic agents *(46% vs. 38%) and *Comorbidities and polypharmacy *(33% vs. 19%), both including safety-related issues, were selected significantly (p < 0.001) more often by GPs. No between-group difference was observed for the *Patient-related reasons *category. The GP-reported HbA_1c _threshold for initiating antihyperglycaemic therapy was significantly (p < 0.001) lower for younger patients (mean ± standard deviation: 7.3% ± 0.7) compared to older patients (7.5% ± 0.9).

**Conclusions:**

GPs selected reasons related to *Mild hyperglycaemia *for non-treatment of their untreated patients with newly diagnosed type 2 diabetes mellitus, despite nearly one-third of these patients having their most recent HbA_1c _value ≥7%. The findings further suggest that safety-related issues may influence the non-treatment of older patients with type 2 diabetes mellitus.

## Background

In 2009, the prevalence of diabetes was 4% in the United Kingdom (UK), with the number of people diagnosed with diabetes increasing from 1.4 million in 1996 to 2.6 million [1]. This estimate is projected to reach 4 million by 2025. Most of these patients will be diagnosed with type 2 diabetes mellitus due to the increasing population of older adults and prevalence of obesity. At the time of diagnosis of type 2 diabetes mellitus, initiating treatment with metformin along with lifestyle changes is recommended for most adults without special considerations for older people [2]. NICE (National Institute of Health and Clinical Excellence) recommends initiating metformin therapy in UK patients with type 2 diabetes mellitus after a period of lifestyle modification [3].

Approximately 40% of patients with diagnosed type 2 diabetes mellitus remain untreated with antihyperglycaemic agents despite having inadequate glycaemic control [4-7]. Some studies have shown that older patients with newly diagnosed type 2 diabetes mellitus are less likely to receive antihyperglycaemic therapy [8,9]. Furthermore, although older patients appear to have improved therapeutic outcomes if antihyperglycaemic therapy is intensified compared to younger patients, younger patients are more likely to receive therapy intensification [10]. Others have reported that age does not influence the initiation or intensification of antihyperglycaemic therapy [4,11]. Nonetheless, delayed treatment initiation or intensification may have significant negative outcomes for older patients who typically present with greater medical comorbidity and vulnerability to untreated hyperglycaemia. Therefore, it was important to understand the reasons of general practitioners (GPs) for not treating younger (< 65 years) and older (≥65 years) patients with newly diagnosed type 2 diabetes mellitus with antihyperglycaemic agents.

## Methods

### Physician and Patient Selection and Data Collection

A survey was conducted in a sample of GPs from the UK between November 2009 and January 2010. GPs treating patients with type 2 diabetes mellitus in usual care settings across the UK were the primary target group. The GPs originated from the Kantar Health Physician Panel containing over 6, 000 GPs from which a random sample was invited to take part in the survey. All participating physicians had to fulfill the following screening criteria in order to be eligible to take the survey: specialty being general practice, at least two years in practice, at least 50% of professional time spent on direct patient care, be primarily responsible for the diabetes management of at least ten patients per month, and had no participation in a physician's panel activity in the last two months.

Additionally, the GPs must have available patient records of at least one patient from each age category (< 65 or ≥65 years at the time of type 2 diabetes mellitus diagnosis). Each eligible and participating GP provided data from chart review for individual patients who met the following inclusion criteria: at least 18 years of age at time of type 2 diabetes mellitus diagnosis, received no treatment with antihyperglycaemic agents for at least six months following diagnosis and also remained untreated at the time of the survey, and had at least one GP office visit for management of their diabetes within the six months prior to the survey. The six-month time period following diagnosis was selected to allow for recommended lifestyle interventions as initial treatments. GPs were requested to provide results for younger (< 65 years) and older (≥65 years) patients in a 1:1 ratio. GPs could provide results for more than one pair of younger and older patients. The following data were extracted from the patient's charts: demographics, comorbidities, medication use, laboratory measurements, and vital signs. HbA_1c _and fasting blood glucose data were collected at the time closest to diagnosis and time closest to the survey date (i.e., most recent laboratory measurements). Remaining data were collected only at the time closest to the survey date (lipid levels, serum creatinine, estimated glomerular filtration rate, height, weight, and blood pressure). Body mass index was calculated as weight in kilograms divided by squared height in meters. GPs provided relevant patient data via the internet using an electronic data capture form. The target patient sample size was 2, 000 patients, with 1, 000 each for younger and older patients. Approximately 4, 000 GPs were invited to participate. Of the 669 respondents who passed the initial screening questions, 571 GPs were considered eligible to participate. Of the eligible GPs, 358 participated and completed the survey and provided select patient results before the planned sample size was achieved.

According to the guidelines developed by the National Research Ethics Service (NRES: http://www.nres.npsa.nhs.uk/applications/guidance/research-guidance/?entryid62=66984), the present study did not require Research Ethics Committee review for the following reasons: it was a non-randomized physician survey including a brief retrospective chart review conducted by the participating physicians and did not involve any contact with patients or interventions. Furthermore, patients and participating physicians were both completely de-identified in data collection and analysis.

### General Practitioners' Reasons for Non-treatment

The survey was developed based on extensive interviews with an expert panel of practicing physicians and academic researchers. Interviews included discussions on treating older versus younger patients and scenarios where patients are not treated with antihyperglycaemic agents for at least six months after initial diabetes diagnosis and potential reasons relevant to non-treatment with antihyperglycaemic agents. After the survey was drafted according to the experts' opinions, it was presented back to the experts for review and approval. A comprehensive list of 36 possible reasons for non-treatment with antihyperglycaemic agents was compiled. The 36 reasons were grouped into four high-level categories: *Mild hyperglycaemia *(three items), *Factors related to antihyperglycaemic agents *(eighteen items), *Comorbidities and polypharmacy *(five items), and *Patient-related reasons *(ten items). The list was provided to GPs using an electronic data capture form. GPs selected all applicable reasons why their patients had not been treated with antihyperglycaemic agents after diagnosis, and then ranked the reasons in order of importance. In addition, the GPs were asked to provide an HbA_1c _threshold value for initiating antihyperglycaemic therapy for each patient.

### Statistical analyses

Descriptive statistics were used to summarise patient demographics, disease characteristics, and reasons for non-treatment with antihyperglycaemic agents. Reasons for non-treatment were evaluated based on all reported reasons (all-reasons analyses) and the first-ranked reason (first-ranked reasons analyses). The between-age group comparisons were analyzed with t-tests or nonparametric tests for continuous data and χ^2 ^test for categorical data.

## Results

### Patient Characteristics

GPs provided data for 2, 028 patients who were newly diagnosed with type 2 diabetes mellitus and also untreated with antihyperglycaemic therapy for at least six months following diagnosis: 1, 023 younger patients (< 65 yrs; mean age at diagnosis = 48.2 years) and 1, 005 older patients (≥65 years; mean age at diagnosis = 70.1 years) (Table 1). Compared with younger patients, older patients had a longer duration of type 2 diabetes mellitus (i.e., time from first diagnosis of type 2 diabetes mellitus to the survey date), a lower body mass index, and higher prevalence of cardiovascular conditions and microvascular complications (especially renal disease) and were taking a greater number of medications (all p < 0.001; Table 1). A higher proportion of younger patients was male and lived independently (Table 1). Measurements related to glycaemic control did not differ between age groups (Table 1). Although HbA_1c _tended to decline from baseline (i.e., difference between measurement closest to diagnosis and most recent measurement) in both groups, the proportion of patients with their most recent measure of HbA_1c _≥7% was approximately 31% and not different between younger and older patients (Table 1).

**Table 1:** Characteristics of patients provided by the general practitioners

	Patients < 65 years (n = 1, 023)	Patients ≥ 65 years (n = 1, 005)	p-value
**Demographics**			

Age at survey, years	50.7 ± 9.2	74.1 ± 7.3	< 0.001

Male	61	54	0.001

Body mass index, kg/m^2^	31.2 ± 6.4	28.9 ± 5.4	< 0.001

**Diabetes-related characteristics**			

Age at diabetes diagnosis, years	48.2 ± 9.2	70.1 ± 8.5	--

Duration of diabetes*, months	18 (9, 36)	25 (11, 60)	< 0.001

HbA_1c _closest to diabetes diagnosis, %	7.4 ± 1.1	7.4 ± 1.2	0.389

Most recent HbA_1c_, %	6.8 ± 0.9	6.8 ± 0.7	0.101

HbA_1c _change from diagnosis, %	-0.5 (-6.9, 7.5)	-0.4 (-8.5, 1.5)	0.189

Proportion with most recent HbA_1c _≥7%	31	32	0.600

FBG closest to diagnosis, mmol/L	9.1 ± 2.8	9.0 ± 2.6	0.505

Most recent FBG, mmol/L	7.1 ± 1.9	7.0 ± 2.0	0.824

Proportion with most recent FBG ≥7 mmol/L	21	20	0.380

**Other characteristics and comorbidities**			

Serum creatinine, μmol/L	85.7 ± 25.5	97.7 ± 31.7	< 0.001

LDL-cholesterol, mmol/L	2.7 ± 2.3	2.5 ± 1.3	0.019

HDL-cholesterol, mmol/L	1.3 ± 0.5	1.4 ± 0.5	0.084

Triglycerides, mmol/L	2.1 ± 1.3	1.7 ± 0.8	< 0.001

Cardiovascular conditions	5.2	18.3	< 0.001

Ischemic heart disease	3.5	9.4	< 0.001

Myocardial infarction	1.8	4.4	< 0.001

Peripheral vascular disease	0.9	2.1	0.027

Stroke	0.6	3.9	< 0.001

Microvascular complications	4.3	15.4	< 0.001

Neuropathy	0.7	1.3	0.184

Retinopathy	1.1	2.0	0.105

Renal disease	2.8	13.2	< 0.001

Total number of medications	2 (0, 3)	3 (1, 6)	< 0.001

Patient's living situation			

Living with spouse/other family member/alone	98.8	89.1	< 0.001

Data are expressed as mean ± standard deviation, frequency (%), or median (interquartile range).

FBG = fasting blood glucose

*Time from first diagnosis of type 2 diabetes mellitus to survey date

### Survey Results

Collectively, reasons within the *Mild hyperglycaemia *category were chosen more frequently relative to those in the other categories by GPs as reasons for non-treatment in both age groups, with no significant differences between age groups in the all-reasons analysis (Table 2). *Factors related to antihyperglycaemic agents *were selected more often (p < 0.001) for older patients compared with younger patients. Within this category, numerous individual reasons accounted for the between-group difference including some related to side effects (e.g., hypoglycaemia, fluid retention, and fracture) and others related to the cognitive or physical function of the patient (Table 2). Reasons related to *Comorbidities and polypharmacy *were also selected significantly more often (p < 0.001) for older patients. Within this category, disease or medication burdens and factors related to polypharmacy (side effects or drug-drug interactions) were selected as reasons with greater frequency in the older patients. No differences overall were observed in the *Patient-related reasons *category. However within this category, fear of weight gain was chosen by GPs more often for younger patients, whereas non-significant trends were observed for more GPs selecting fear of hypoglycaemia and physical difficulty in taking medications as reasons for non-treatment in older patients (Table 2).

**Table 2:** All reasons selected by general practitioners for non-treatment of patients with type 2 diabetes mellitus

Reasons, n (%)	Patients < 65 years n = 1, 023	Patients ≥65 years n = 1, 005	p-value
**Mild Hyperglycaemia**	**897 (87.7)**	**866 (86.2)**	**0.323**

HbA_1c _value stable, drug therapy not necessary	278 (27.2)	264 (26.3)	0.652
HbA_1c _value close to NICE recommended threshold	316 (30.9)	326 (32.4)	0.474
Blood glucose values under control with diet and exercise	301 (29.4)	330 (32.8)	0.103

**Factors Related to Antihyperglycaemic Agents**	**386 (37.7)**	**463 (46.1)**	**< 0.001**

May cause hypoglycaemia	176 (17.2)	250 (24.9)	< 0.001
May cause fluid retention	67 (6.6)	98 (9.8)	0.009
May cause weight gain	173 (16.9)	157 (15.6)	0.435
May cause gastrointestinal side effects	203 (19.8)	200 (19.9)	0.999
May increase risk of fracture	45 (4.4)	68 (6.8)	0.026
May increase cardiovascular risk	49 (4.8)	68 (6.8)	0.057
May increase risk of lactic acidosis	78 (7.6)	96 (9.6)	0.132
Uncertainty how to dose certain drug	26 (2.5)	25 (2.5)	0.999
Not clear if several agents are safe	30 (2.9)	34 (3.4)	0.612
Efficacy of agents not clear	30 (2.9)	24 (2.4)	0.492
Safety of agents not clear	34 (3.3)	30 (3.0)	0.704
Primary Care Trust cost concerns	24 (2.4)	26 (2.6)	0.776
Cognitive burden of therapy administration too high for patient	66 (6.5)	121 (12.0)	< 0.001
Cognitive burden of monitoring glucose too high for patient	48 (4.7)	87 (8.7)	< 0.001
Difficulties/ability to change patient's lifestyle	118 (11.5)	119 (11.8)	0.836
Risk of non-compliance (not related to side effects)	115 (11.2)	132 (13.1)	0.198
Risk of non-compliance due to side effects	80 (7.8)	115 (11.4)	0.007
Lack of monitoring due to physical limitations (e.g., dexterity)	32 (3.1)	82 (8.2)	< 0.001

**Comorbidities and Polypharmacy**	**192 (18.8)**	**333 (33.1)**	**< 0.001**

Patient has other severe disease(s)	69 (6.7)	172 (17.1)	< 0.001
Medical diabetes treatment is contraindicated	27 (2.6)	38 (3.8)	0.166
Patient is taking several other medications already	108 (10.6)	216 (21.5)	< 0.001
Risk of side effects (related to polypharmacy)	124 (12.1)	191 (19.0)	< 0.001
Risk of drug-drug interactions (related to polypharmacy)	60 (5.9)	100 (10.0)	< 0.001

**Patient-Related Reasons**	**443 (43.2)**	**416 (41.4)**	**0.419**

Patient denial/anger/depression related to diabetes diagnosis	120 (11.7)	108 (10.8)	0.527
Patient's follow-up visit is overdue	92 (9.0)	64 (6.4)	0.030
Patient does not want to take (additional) medication	325 (31.8)	305 (30.4)	0.502
Fear of hypoglycaemia	71 (6.9)	93 (9.3)	0.061
Fear of weight gain	112 (11.0)	78 (7.8)	0.015
Fear to change from diet/exercise to oral agents	100 (9.8)	88 (8.8)	0.445
Fear to change from diet/exercise to insulin	54 (5.3)	53 (5.3)	0.999
Patient has physical difficulty taking medication	29 (2.8)	44 (4.4)	0.073
Drug therapy decreases quality of life	61 (6.0)	76 (7.6)	0.158

**Figure 1:**
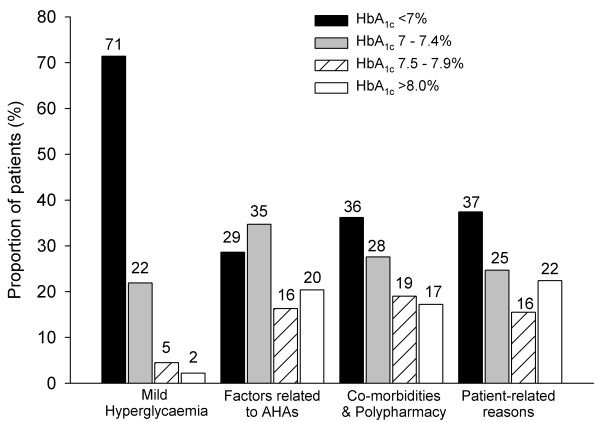
**The distribution of the most recent HbA_1c _level by GP's first-ranked reason for non-treatment**. AHAs = antihyperglycaemic agents.

In an analysis using only first-ranked reasons, no significant differences within the high-level reasons categories were observed between age groups. With age groups combined, the overall percentage of GPs selecting reasons within the *Mild hyperglycaemia *category as the first-ranked reason was 79% followed by *Patient-related reasons *(14%), *Comorbidities and polypharmacy *(4%) and *Factors related to antihyperglycaemic agents *(3%). The GPs' first-ranked reasons for non-treatment were also evaluated by category and most recent HbA_1c _stratum. HbA_1c _level was associated with selecting reasons within *Mild hyperglycaemia *category (Figure 1). GPs selected reasons within *Mild hyperglycaemia *category for 29% of their patients who had an HbA_1c _≥7%.

### GP-reported HbA_1c _threshold for initiating an antihyperglycaemic agent

GP-reported HbA_1c _threshold for initiating antihyperglycaemic therapy was significantly (p < 0.001) lower for younger patients (mean ± standard deviation: 7.3% ± 0.7) compared to older patients (7.5% ± 0.9). The proportion of patients with their most recent HbA_1c _measurement above their GP-reported HbA_1c _threshold was significantly higher (p = 0.002) in the younger patients (14.3%) compared to the older patients (10.4%).

## Discussion

This study assessed the reasons reported by a sample of UK GPs for not initiating antihyperglycaemic therapy in younger (< 65 years) and older (≥65 years) patients with newly diagnosed type 2 diabetes mellitus who remained untreated for at least six months following the initial diagnosis. In patients whose HbA_1c _was well controlled at the time of the survey, the GPs reason for not initiating treatment was often that the patients had only *Mild hyperglycaemia*, whereas for less well-controlled patients, other reasons were more prominent. Interestingly, 29% of patients had an HbA_1c _≥7% despite their GP's selection of the first-ranked reason being *Mild hyperglycaemia*. In a cross-sectional survey study of US-based practices, physicians provided reasons of "improving/doing well" for over 50% of the patients who had an HbA_1c _≥7% and no action taken on therapy (i.e., lifestyle and/or medication) after a recent office visit [12]. These findings represent an important gap between treatment guidelines and the current clinical practice.

GPs were also requested to provide an HbA_1c _threshold for initiating antihyperglycaemic therapy for individual patients included in this study. The mean for the GP-reported HbA_1c _threshold for initiating antihyperglycaemic therapy was lower for younger patients than for older patients (7.3% vs. 7.5%). This lower threshold for younger patients may explain why a higher proportion of younger patients (14%) had their most recent HbA_1c _value exceed their GP-defined individualized threshold for initiating treatment than that of the older patients (10%). Interestingly, the GP-reported thresholds on average were near the HbA_1c _value of 7.5%, which was recently associated with the lowest incidence of all-cause mortality and progression to macrovascular events [13]. NICE recommends treatment targets between 6.5% and 7.5% depending on the extent of pre-existing comorbid conditions and agreement with the patient [3]. However, GPs actually applied an HbA_1c _threshold ≥7.5% for initiating antihyperglycaemic therapy in about half of these older patients.

Reasons within the categories of *Factors related to antihyperglycaemic agents *and *Comorbidities and polypharmacy *were selected more frequently by GPs as reasons for non-treatment of older patients. The individual reasons with significant between-group differences appeared to be focused on issues related to safety (e.g., risk of side effects or drug-drug interactions), disease or medication burdens for patients and cognitive or physical function of the patient. Although not assessed for age-related differences, similar reasons have been provided by physicians for their patients not receiving action in diabetes treatment despite having elevated HbA_1c _[12]. Diabetes therapies that have demonstrated efficacy and safety in patients, especially older patients, may allay some of these concerns raised by GPs. Furthermore, GPs in the present study set a higher HbA_1c _threshold for initiating treatment in older patients. These results are consistent with previous studies that have shown that older patients with newly diagnosed type 2 diabetes mellitus were less likely to receive antihyperglycaemic therapy [8,9]. The reasons identified in the present study may help explain the clinical inertia or inequality of health care observed in previous studies. It is understandable to focus on issues related to safety and functional capacity when considering diabetes treatments and management in older patients, especially frail patients [14]. However, in the present study, a majority of older patients lived independently. Furthermore, given that older patients have higher prevalence of comorbidities, failure to treat or delays in diabetes treatment for this population may have greater health implications than for younger patients.

There are many factors that influence the treatment of patients with type 2 diabetes mellitus. In a focus group setting with family physicians, Brown et al. [15] identified patient-, physician- and systemic-related factors that were considered barriers and facilitators to the management of patients with type 2 diabetes mellitus. For patient factors, physicians felt that many patients with type 2 diabetes mellitus failed to recognise the seriousness of their disease due its asymptomatic nature. Education was seen as both a barrier and facilitator to disease management. Physicians indicated that early education led to better outcomes for their patients. Conversely, physicians felt overwhelmed with all of the different treatment guidelines for their diabetic patients and associated comorbid conditions [15]. In another study, reasons of GPs for not prescribing lipid-lowering agents in patients with type 2 diabetes mellitus were divided into patient- and physician-related factors [16]. Patient-related factors included compliance or refusal to take prescribed medication due to expected or perceived side effects. Physician reasons were related to proximity to treatment targets, perceived lack of benefits in patients with short life expectancy and expected compliance problems with their patients [16]. In the present study, risk of non-compliance was selected by up to 13% of GPs as a reason for non-treatment. Overall, these and the present results demonstrate that diverse factors influence the GP's decision to initiate treatment in patients with type 2 diabetes mellitus.

The following limitations should be considered when interpreting the results of this study. Observed trends pertain to GPs and might not be generalisable to endocrinologists, diabetes, or GPs who do not treat many patients with type 2 diabetes mellitus. A selection bias may have occurred as the GPs needed to meet specific criteria in order to be eligible to participate, which may have limited the participating GPs to those who had a greater focus on diabetes care. Other reasons not identified may influence GPs for not initiating therapy (e.g., life expectancy, overall quality of life, or recent clinical findings). All physician-reported laboratory measures closest to diagnosis were included in the analysis regardless of the timing of measurement. Although GPs provided the clinical data of patients who met specific criteria, the GPs self selected the patients from their practices. GPs entered select patient data in the online form and completeness of the form was assessed. Validation of data extraction was confirmed using built-in logical checks of the data (e.g., edit checks).

## Conclusions

GPs selected reasons related mainly to *Mild hyperglycaemia *for non-treatment with antihyperglycaemic agents for their untreated patients with newly diagnosed type 2 diabetes mellitus, despite nearly one-third of these patients having their most recent HbA_1c _≥7%. In addition, the survey findings suggest that issues related to safety of antihyperglycaemic agents, burden to the patients, and cognitive or physical function of the patient influence the non-treatment of older patients with type 2 diabetes mellitus.

## Competing interests

QZ, EM, KJ, MJD, and LR are employees of Merck Sharp & Dohme Corp. SM and AJS have no conflicts related to this analysis.

## Authors' contributions

QZ, EM, KJ, LR, SM, and AS were involved in the concept and design of the study. QZ, EM, KJ, and SM were involved in the data collection and/or analysis. All authors were involved in interpretation of the results. MJD and EM drafted the article and all authors were involved in the critical revision and approval of the article.

## Pre-publication history

The pre-publication history for this paper can be accessed here:

http://www.biomedcentral.com/1472-6823/11/17/prepub
